# Norman Lloyd Price

**Published:** 1945

**Authors:** 


### NORMAN LLOYD PRICE, M.D., M.R.C.P.

THE medical profession in Bristol has suffered a severe loss in the death, at the age of 38, of one of its younger and most able physicians, Dr. Norman Lloyd Price, M.D., M.R.C.P. Second son of the late Rev. W. W. Price, former Minister of Westbury Park Methodist Church, he graduated at Bristol University, where he took his M.B.

For some years he was Deputy M.O. at Southmead Hospital, his tenure covering the early days of the war, when he did valuable work during the blitz period under the Emergency Medical Services scheme. Subsequently he became Honorary Physician at the Children's Hospital, after which he entered private practice, specialising as a consultant for children. He was also a voluntary consultant at Bristol Dispensary, Redcross Street.

At all the institutions with which he was connected he was popular alike with the staff and the patients, who greatly appreciated his consideration and kindliness.

He enjoyed a game of golf, and with a longish handicap on one occasion won the Corporation Staff Cup at Failand.

His death followed an illness lasting 21 years. He leaves a widow, to whom we offer our sympathy.

